# The relation between mortality from malignant melanoma and early detection in the Cancer Research Campaign Mole Watcher Study

**DOI:** 10.1054/bjoc.2001.2012

**Published:** 2001-09

**Authors:** J Melia, S Moss, D Coleman, T Frost, R Graham-Brown, J A A Hunter, R A Marsden, A du Vivier, A P Warin, J White, S M Whitehead, M A Wroughton

**Affiliations:** Cancer Screening Evaluation Unit, Institute of Cancer Research, Section of Epidemiology, D Block, Cotswold Road, Sutton, Surrey SM2 5NG; Dept. Dermatology, Royal Devon & Exeter Hospital, Barrack Road, Exeter, EX2 5DW; Dept. Dermatology, Leicester Royal Infirmary, Infirmary Square, Leicester, LE1 5WW; Dept. Dermatology, The Royal Infirmary, Level 4, Phase 1, Edinburgh, EH3 9YW; Dept. Dermatology, St. George's Hospital, Blackshaw Road, London, SW17 0QJ; Dept. Dermatology, King's College Hospital, Denmark Hill, London, SE5 9RS; Dept. Dermatology, Royal South Hants Hospital, Graham Road, Southampton, SO9 4PE; Dept. Public Health Medicine, South Derbyshire HA, Boden House, Main Centre, Derby, DE1 2PH; 30 Royal Standard House, Standard Hill, Nottingham, NG1 6FX, UK

**Keywords:** melanoma, early detection, mortality

## Abstract

Between 1987 and 1989 the Cancer Research Campaign funded a health education programme for the early detection of cutaneous malignant melanoma in the general population in 6 health districts of England and 1 health board in Scotland (population of 3 million). The intervention was evaluated by studying its effects on annual and cumulative mortality rates for melanoma. Population-based data on mortality from melanoma were collected in the intervention areas, the health regions covering those areas, and 5 other health regions of England from 1981 to 1996. Deaths from melanoma in cases diagnosed after the start of the intervention were used to study cumulative mortality rates. The annual mortality rates for melanoma, 1981 to 1996, showed no significant difference in their trends between the intervention areas, and other areas of England and Wales. After adjustment for pre-intervention rates, there was also no significant reduction in cumulative mortality from melanoma in the intervention areas compared with the non-intervention areas: rate ratio 1.2 (95% Cl 0.9–1.7) in men, 0.9 (95% Cl 0.7–1.3) in females. The lack of a significant reduction in melanoma mortality associated with the intervention raises questions about this approach to early detection and emphasises the need for new strategies. © 2001 Cancer Research Campaignhttp://www.bjcancer.com


*British Journal of Cancer* (2001) **85**(6), 803–807
© 2001 Cancer Research Campaign
doi: 10.1054/ bjoc.2001.2012, available online at http://www.idealibrary.com on IDEAL

### The relation between mortality from malignant melanoma and early detection in the Cancer Research Campaign Mole Watcher Study

**J Melia[1], S Moss[1], D Coleman[1], T Frost[2], R Graham-Brown[3], JAA Hunter[4], RA Marsden[5], A du Vivier[6], AP Warin[2], J White[7]*, SM Whitehead[8] and MA Wroughton[9]**

[1]Cancer Screening Evaluation Unit, Institute of Cancer Research, Section of Epidemiology, D Block, Cotswold Road, Sutton, Surrey SM2 5NG; [2]Dept. Dermatology, Royal Devon & Exeter Hospital, Barrack Road, Exeter EX2 5DW; [3]Dept. Dermatology, Leicester Royal Infirmary, Infirmary Square, Leicester LE1 5WW; [4]Dept. Dermatology, The Royal Infirmary, Level 4, Phase 1, Edinburgh EH3 9YW; [5]Dept. Dermatology, St. George's Hospital, Blackshaw Road, London SW17 0QJ; [6]Dept. Dermatology, King's College Hospital, Denmark Hill, London SE5 9RS; [7]Dept. Dermatology, Royal South Hants Hospital, Graham Road, Southampton SO9 4PE; [8]Dept. Public Health Medicine, South Derbyshire HA, Boden House, Main Centre, Derby DE1 2PH; [9]30 Royal Standard House, Standard Hill, Nottingham NG1 6FX, UK

**Summary** Between 1987 and 1989 the Cancer Research Campaign funded a health education programme for the early detection of cutaneous malignant melanoma in the general population in 6 health districts of England and 1 health board in Scotland (population of 3 million). The intervention was evaluated by studying its effects on annual and cumulative mortality rates for melanoma. Population-based data on mortality from melanoma were collected in the intervention areas, the health regions covering those areas, and 5 other health regions of England from 1981 to 1996. Deaths from melanoma in cases diagnosed after the start of the intervention were used to study cumulative mortality rates. The annual mortality rates for melanoma, 1981 to 1996, showed no significant difference in their trends between the intervention areas, and other areas of England and Wales. After adjustment for pre-intervention rates, there was also no significant reduction in cumulative mortality from melanoma in the intervention areas compared with the non-intervention areas: rate ratio 1.2 (95% Cl 0.9–1.7) in men, 0.9 (95% Cl 0.7–1.3) in females. The lack of a significant reduction in melanoma mortality associated with the intervention raises questions about this approach to early detection and emphasises the need for new strategies. © 2001 Cancer Research Campaign http://www.bjcancer.com

**Keywords**: melanoma; early detection; mortality

From 1987 to 1989 the Cancer Research Campaign (CRC) ran a health education programme to promote the early detection of cutaneous malignant melanoma in the general population in 6 district health authorities (DHAs) in England and 1 health board (HB) in Scotland (Melia et al, 1995a, 1995b). This followed a similar intervention in the west of Scotland (MacKie and Hole, 1992) involving the local media, leaflets and posters. The Scottish intervention was reported to have led to a reduction in mortality from melanoma in women, but not men, following a decrease in the incidence rate for thick melanomas (Breslow ≥ 3.5 mm).

Results from 2 CRC study areas did not indicate that the intervention had led to a reduction in the incidence rate of thick melanomas (Whitehead et al, 1989; Healsmith et al, 1994; Herd et al, 1995). However, the follow-up period was only up to 3 years, and the value of using thick melanomas as a surrogate measure for melanoma mortality is uncertain.

The purpose of this paper is to report the effects of the intervention on annual and cumulative mortality rates for melanoma. The advantage of the latter is that it excludes deaths in cases diagnosed before the start of the intervention which could not therefore have benefited (Tabar et al, 1985; Moss et al, 1999). The rates were compared between the intervention areas and 2 groups of comparison areas elsewhere in England and Scotland, with data collected up to 9 years after the intervention. In addition, trends in the annual mortality rates for melanoma were compared between the 3 groups of areas and with England and Wales, similar to the analyses conducted in Scotland.

*Received 1 December 2000*
*Revised 11 June 2001*
*Accepted 3 July 2001*

*Correspondence to:* J Melia

### METHODS

### The health education programme

The intervention used the CRC Mole Watcher leaflet (Whitehead et al, 1989; Williams et al, 1990; Healsmith et al, 1994; Herd et al, 1995) which provided advice about the early signs of melanoma based on a 7 point checklist (MacKie, 1989), and encouraged early reporting of suspicious lesions to general practitioners (GPs). The leaflet did not advise on regular self-screening. The main thrust of the intervention was in the summer of 1987, and it was then maintained with some variability until the summer of 1989. The intervention areas had been selected because local hospital-based registers already existed to collect information on melanoma. The CRC funded a pigmented lesion clinic (PLC) in each area from 1987 to 1989 to help with the increase in hospital referrals, but dermatology clinics also continued to receive referrals (Osborne et al, 1998).

* Dr White sadly died before this paper was accepted for publication.

**Table 1** Population size[a] and annual melanoma mortality rate, age adjusted to the European population, at the beginning of the study period (taking a 3-year average from 1981–83) in males and females

| Groups of areas | Population size* (millions) | Mortality | |
|---|---|---|---|
| | | Rate per $10^5$ | 95% CI |
| Males | | | |
| Non-intervention areas | | | |
| Regions away from intervention | 6.7 | 1.85 | 1.66–2.05 |
| Rest of intervention region | 8.1 | 1.87 | 1.70–2.06 |
| Intervention areas | 1.4 | 1.92 | 1.52–2.39 |
| Females | | | |
| Non-intervention areas | | | |
| Regions away from intervention | 6.8 | 1.87 | 1.68–2.06 |
| Rest of intervention region | 8.3 | 1.70 | 1.54–1.87 |
| Intervention areas | 1.5 | 1.43 | 1.09–1.83 |

[a]Population in 1987 at start of intervention aged 15 to 74 years.

### Study populations

The age at death in the analyses was restricted to 15 to 74 years because of the low incidence of melanoma at younger ages, and increased risk of competing causes of death at older ages, respectively. Results were compared between the 7 intervention areas, areas in the same regional health authorities as the intervention areas (intervention regions), and 5 other regional health authorities (non-intervention regions). The regions selected for the last group were chosen because, at the time of data collection, their cancer registries could provide up-to-date data for the study period. The population sizes are given in Table 1, and details of the study areas in the Appendix.

### Data collection on melanoma deaths

The Office for National Statistics (ONS) and the Scottish Health Service Information and Statistics Division (ISD) provided aggregated data on melanoma mortality (ICD 172) by sex, age and area of residence for the calculation of annual mortality rates from 1981 to 1996.

To produce cumulative mortality rates, date of diagnosis is necessary. The latter is not available from routinely produced aggregated data so individual data were collected from cancer registries. Each registry used their own coding system for cause of death. From these systems, the underlying cause of death was determined. Quality control checks on the coding of death by the registries in the intervention areas were conducted by comparing their codes with a random sample of death certificates supplied by ONS. Agreement in general was good, but in a very small number, the death may have been coded as the underlying cause where it was mentioned in Part 1 of the death certificate but was not the underlying cause. Where a date of death was recorded, but no cause given, copies of death certificates were obtained from ONS.

Completeness of data on deaths for both analyses was expected to be high because it depended on the death registration system and the routine notification of cancer deaths by ONS to the cancer registries. No checks on completeness were made, but it is unlikely to have varied greatly between regions.

### Outcome measures

The annual mortality rate (the number of deaths in each year divided by the population in that year) does not take time of diagnosis into account and so it includes both people who were diagnosed before and after the intervention. The cumulative mortality excludes these people, restricting deaths to people who were diagnosed during the intervention. However, early detection brings forward the date of diagnosis (lead-time bias) for some people which would otherwise have been diagnosed after the end of the intervention. Consequently, a longer period than the intervention must be used although how long depends on the lead time, which is unknown. Deaths from the start of the campaign until the end of 1991 (a lead time of 2 years) have been included. The denominator for the cumulative mortality is the population who were exposed to the campaign which is assumed to be the population in 1987.

### Statistical analyses

Analyses were conducted on the full dataset, and on subgroups: those aged < 65 because the effects of early detection may be greater at younger ages (MacKie and Hole, 1992), and excluding Scottish areas because the 1985 intervention may have affected trends in melanoma. The effect of distance from the intervention areas was initially studied by comparing areas that were adjacent and non-adjacent to the intervention areas in those regions. However, no consistent or significant trends were found so the intervention regions were taken as one group. The number of deaths, offset by the population, was analysed in Poisson regression on both annual and cumulative mortality, separately for each sex with an age term (in 10-year age bands).

In the analyses of the annual mortality rates, within each group of areas, an additional variable (i) was created which estimated the change in the slope in 1988–96 relative to that in 1981–87. To compare the change in trends in different groups of areas, an interaction term (i * area) was studied in a model including all groups of areas. Different cut off points were studied.

For the cumulative mortality, the rate ratios are expressed relative to the non-intervention regions and to pre-intervention mortality. Pre-intervention mortality for the years 1984 to 1986 was compiled from national statistics. The interaction between the groups of areas and pre-and post-intervention period (1984–86 and 1987–96, respectively) was used to study the effect of the intervention. A test for heterogeneity between the 7 intervention areas was studied by treating each intervention area as an individual

 *© 2001 Cancer Research Campaign*

study area after adjusting for the pre-intervention rates (Sharp and Sterne, 1997).

### RESULTS

### Annual melanoma mortality rates

There was no significant difference in the annual mortality rate for melanoma in males or females, age adjusted to the European population (Office for National Statistics, 1998), between the groups of areas at the start of the study period, 1981–1983 (Table 1). The annual mortality rates, age adjusted to the European population (Figures 1 and 2) appeared to show a decrease in melanoma mortality for women in all groups of areas after 1993, and were similar to the overall trend for England and Wales. In regression analyses, changes in the slope of the graphs were studied using an interaction term as described in the Methods. Different cut-off periods from 1988 onwards were used and adjustments made for any differences in trends between groups of areas before each cut-off period e.g. 1981 to 1987. No significant differences were found in the trends between areas (*P* > 0.30). Similar results were found when restricted to people aged < 65 and to only those in England.

### Cumulative melanoma mortality rates

The age-adjusted cumulative mortality rates, 1987 to 1996, in the intervention areas, unadjusted for pre-intervention mortality rates between the areas, showed no significant difference between groups of areas (*P* > 0.30 for males and females, Table 2). After adjusting for pre-intervention rates, there remained no significant

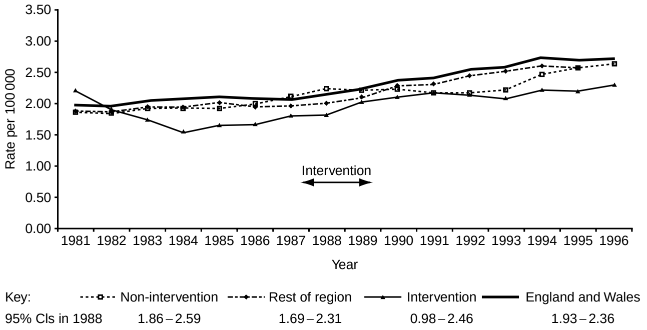

**Figure 1** Annual mortality rate from melanoma in males age adjusted to the European population expressed as a three year moving average

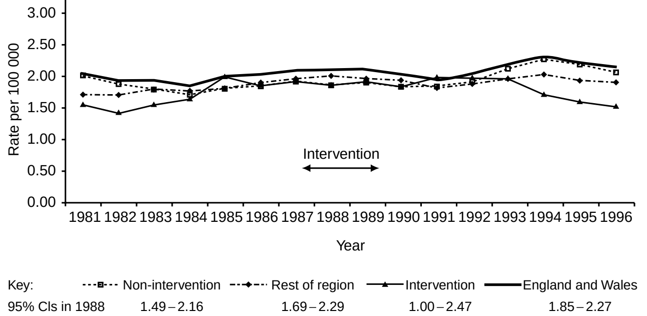

**Figure 2** Annual mortality rate from melanoma in females age adjusted to the European population expressed as a three year moving average difference in rates for the intervention areas (*P* > 0.30 for males and females separately) and the rest of the intervention regions (*P* > 0.40 for males and females separately) compared with the regions away from the intervention. Similar results were found after adjusting for the pre-intervention rates when restricted to people aged < 65 and to only those in England. The results also did not vary with different lead times.

The heterogeneity test showed no significant difference in the effect of the intervention on melanoma mortality between the 7 intervention areas (*P* = 0.30 and *P* = 0.45 for males and females, respectively).

### DISCUSSION

The CRC early detection intervention of 1987–89 did not result in a significant reduction in the annual or cumulative mortality rates for melanoma. Any large effect on melanoma mortality from the intervention should have been detected. For example, based on the observed cumulative mortality in the non-intervention areas, the study had 80% power to detect a 30% reduction in males or a 28% reduction in females in the intervention areas at the 5% significance level. It may be that a longer period of follow-up is required to study an effect on mortality, or the intervention may have been more effective in subgroups of the population which could not be studied because the number of deaths would have been too small.

In this study some undetected benefit from the Mole Watcher intervention cannot be ruled out, but any large effect on mortality should have been seen. As this study was not a randomised controlled trial, the outcome measures of mortality were compared both before and after the start of the intervention between areas exposed to different degrees to the intervention. Limitations associated with the outcome measures, sources of data and exposure to the intervention are discussed.

Cumulative mortality has been used as an outcome measure in randomised controlled trials of screening, and has the advantage over annual mortality rates that it is a count of melanoma deaths only in those cases diagnosed after the start of, and most likely to have benefited from, the intervention (Tabar et al, 1985; Moss et al, 1999) To overcome the problem of lead-time bias, the melanoma cases in which deaths were counted had their period of diagnosis extended beyond the end of the intervention to 1991.

Unlike a screening trial in which data are collected on individuals regarding their screening history, diagnosis and cause of death, it is not feasible in mass media interventions to record for every individual in the target population precisely who has received and acted upon the health education provided. Assumptions have to be made about the population exposed to the intervention. In one survey conducted in association with the Mole Watcher study, awareness of the leaflets and posters increased during the intervention (Melia et al, 1995b), but this does not indicate how well people understood the signs of melanoma or how frequently they examined their skin.

The presence of specialist registers in the intervention areas may indicate a high level of interest in melanoma which may have had some influence on the completeness of records and measure of incidence of melanoma. However, the outcome measure of melanoma mortality is less likely to be biased because of the high ascertainment of mortality data in Britain, and the age group studied were less likely to be affected by competing causes of death than older age groups. The graphs of annual mortality rates

© 2001 Cancer Research Campaign

**Table 2** Cumulative melanoma mortality in 1996 by sex and area

| | Number of deaths 1987–1996 | Cumulative mortality rate per $10^5$ 95% CI | Rate ratio adjusted for age 95% CI | Rate ratio adjusted for age and pre-campaign rates 95% CI |
|---|---|---|---|---|
| Males | | | | |
| Non-intervention areas | | | | |
| Regions away from intervention | 471 | 6.91<br>6.30–7.56 | 1.00 | 1.00 |
| Rest of intervention region | 580 | 7.01<br>6.45–7.60 | 1.14<br>1.02–1.27 | 1.08<br>0.91–1.29 |
| Intervention areas | 99 | 6.71<br>5.46–8.17 | 0.99<br>0.81–1.22 | 1.18<br>0.85–1.65 |
| Females | | | | |
| Non-intervention areas | | | | |
| Regions away from intervention | 549 | 8.17<br>7.50–8.88 | 1.00 | 1.00 |
| Rest of intervention region | 754 | 9.37<br>8.71–10.06 | 0.99<br>0.88–1.12 | 0.99<br>0.82–1.18 |
| Intervention areas | 114 | 7.94<br>6.55–9.54 | 0.98<br>0.79–1.22 | 0.91<br>0.66–1.25 |

confirm that the rates in the intervention areas were not higher than those elsewhere.

Finally, contamination of the comparison areas by health education about early detection could have reduced the potential to demonstrate a difference in either cumulative or annual mortality rates between areas. General media interest in early detection is likely to have affected all areas (Melia et al, 1994), but its effectiveness at reaching all sections of the population and reducing melanoma mortality is doubtful without the back-up of local distribution of health education materials.

Following the Scottish intervention conducted in 1985 (MacKie et al, 1997), a reduction in the annual mortality rate for melanoma was reported among women living in the whole of Scotland but this occurred almost immediately after the start of the intervention. Given the good survival for melanoma in the first 5 years, this reduction is unlikely to have resulted from the 1985 intervention. In this paper, the fluctuations in annual mortality are not explained by the combined effect of the Scottish and CRC interventions, as the results were similar when Scotland was first included and then removed from the analyses.

Mortality from melanoma has been falling or reaching a plateau in several countries (Streetly and Markowe, 1995; Giles et al, 1996; MacKie et al, 1997; La Vecchia et al, 1999; Cohn-Cedermark et al, 2000; Severi et al, 2000). In the UK these trends first seemed to start before the interventions in 1985 and 1987 (Streetly and Markowe, 1995; MacKie et al, 1997). These are mostly cohort effects with decreasing rates occurring in the younger cohorts. It is impossible to claim an effect from one specific factor although early detection may well have contributed. In Australia (Giles et al, 1996) a greater reduction in melanoma mortality was observed following an intensive early detection intervention in Queensland in the 1960s than in other states.

The lack of an effect of the intervention on cumulative mortality occurred despite an apparent rise in the incidence rate of thin melanomas in the intervention areas (Melia et al, 1995b). Others have also shown an apparent rise in the rate for detection of thin melanomas, but no evidence of a reduction in mortality (Dennis, 1999; Lipsker et al, 1999; van der Rhee et al, 1999). The rising incidence rates pre-intervention (Whitehead et al, 1989) raise questions about progression and over diagnosis (Edman and Klaus, 2000). A proportion of thin melanomas may be non-progressive (Burton and Armstrong, 1994) or lesions may be over diagnosed as malignant (Cook et al, 1996).

There are inherent difficulties in evaluating high-profile interventions because of the lack of a control population, and uncertainty about the extent to which the target population acted effectively on the advice about early detection. In addition the evaluation study was set up after the intervention had taken place, and there was a lack of data on Breslow thickness which would enhance any study of early detection. Unlike some registries in Australia, and the USA, Breslow thickness is not routinely recorded by all cancer registries in the UK. Careful evaluation of future strategies for early detection or screening is needed to assess the effectiveness of different interventions which should aim to reduce mortality without causing excessive increased workload (Doherty and MacKie, 1988; Burton et al, 1993; Melia et al, 1995a). In the UK targeted screening has been explored (Little et al, 1995; Jackson et al, 1998; Melia et al, 2000) but there are concerns about the accuracy and psychological effects of self-assessment for melanoma risk (Sinclair, 1998). Screening for very high-risk groups with a family history of melanoma might be combined with health education to promote regular skin self-examination in the general population (Berwick et al, 1996). Small scale studies are needed to evaluate whether early detection of melanoma is effective. Only then should a large-scale evaluation study be conducted to investigate the effects on mortality, and overall cost-effectiveness of early detection.

### ACKNOWLEDGEMENTS

The study was funded by the Cancer Research Campaign. We would like to thank our research assistant Mrs Christine Ellis for collection and organising the data, the pathologists Dr A Fletcher and Dr J Theaker, the cancer registries, the Scottish Melanoma Study Group, and the Office for National Statistics for helping us with the supply of data. We also thank Professor Jocelyn Chamberlain and, posthumously, Dr Ruth Ellman who prepared the original study protocol.

 *© 2001 Cancer Research Campaign*

### APPENDIX

**Intervention areas:** Camberwell; Edinburgh; Exeter; Leicester; Merton, Sutton and Wandsworth; Nottingham and Southampton.

**Regions covering the intervention areas:** Scotland (for Scotland, the rest of Scotland, except Glasgow, which had an earlier intervention in 1985, formed the intervention region relative to Edinburgh.), South East Thames, South West Thames, South Western, Trent and Wessex.

**Non-intervention areas:** East Anglia, North East Thames, North West Thames, West Midlands and Yorkshire.

### REFERENCES

Berwick M, Begg CB, Fine JA, Roush GC and Barnhill RL (1996) Screening for cutaneous melanoma by skin self-examination. *J Natl Cancer Inst* **88**: 17–23

Burton RC and Armstrong BK (1994) Recent incidence trends imply a nonmetastasizing form of invasive melanoma. *Melanoma Res* **4**: 107–113

Burton RC, Coates MS, Hersey P, Roberts G, Chetty MP, Chen S, Hayes MH, Howe CG and Armstrong BK (1993) An analysis of a melanoma epidemic. *Int J Cancer* **55**: 765–770

Cohn-Cedermark G, Mansson-Brahme E, Rutqvist LE, Larsson O, Johansson H and Ringborg U (2000) Trends in mortality from malignant melanoma in Sweden, 1970–1996. *Cancer* **86**: 348–355

Cook MG, Clarke TJ, Humphreys S, Fletcher A, McLaren KM, Smith NP, Stevens A, Theaker JM and Melia J (1996) The evaluation of diagnostic and prognostic criteria and the terminology of thin cutaneous malignant melanoma by the CRC Melanoma Pathology Panel. *Histopathol* **28**: 497–512

Dennis LK (1999) Analysis of the melanoma epidemic, both apparent and real: data from the 1973 through 1994 surveillance, epidemiology, and end results program registry. *Arch Dermatol* **135**: 275–280

Doherty VR and MacKie RM (1988) Experience of a public education programme on early detection of cutaneous malignant melanoma. *BMJ* **297**: 388–391

Edman RL and Klaus SN (2000) Is routine screening for melanoma a benign practice? *JAMA* **284**: 883–889

Giles GG, Armstrong BK, Burton RC, Staples MP and Thursfield VJ (1996) Has mortality from melanoma stopped rising in Australia? Analysis of trends between 1931 and 1994. *BMJ* **312**: 1121–1125

Healsmith MF, Bourke JF, Osborne JE and Graham Brown RA (1994) An evaluation of the revised seven-point checklist for the early diagnosis of cutaneous malignant melanoma. *Br J Dermatol* **130**: 48–50

Herd RM, Cooper EJ, Hunter JA, Mclaren K, Chetty U, Watson AC and Gollock J (1995) Cutaneous malignant melanoma. Publicity, screening clinics and survival–the Edinburgh experience 1982–90. *Br J Dermatol* **132**: 563–570

Jackson A, Wilkinson C, Ranger M, Pill R and August P (1998) Can primary prevention or selective screening for melanoma be more precisely targeted through general practice? A prospective study to validate a self administered risk score. *BMJ* **316**: 34–39

La Vecchia C, Lucchini F, Negri E and Levi F (1999) Recent declines in worldwide mortality from cutaneous melanoma in youth and middle age. *Int J Cancer* **81**: 62–66

Lipsker DM, Hedelin G, Heid E, Grosshans EM and Cribier BJ (1999) Striking increase of thin melanomas contrasts with stable incidence of thick melanomas. *Arch Dermatol* **135**: 1451–1456

Little P, Keefe M and White J (1995) Self screening for risk of melanoma: validity of self mole counting by patients in a single general practice. *BMJ* **310**: 912–916

MacKie RM (1989) *Malignant Melanoma. A guide to early diagnosis*. Edinburgh: ??

MacKie RM and Hole D (1992) Audit of public education campaign to encourage earlier detection of malignant melanoma. *BMJ* **304**: 1012–1015

MacKie RM, Hole D, Hunter JAA, Rankin R, Evans A, Mclaren K, Fallowfield M, Hutcheon A and Morris A (1997) Cutaneous malignant melanoma in Scotland: Incidence, survival and mortality 1979–1994. *BMJ* **315**: 1117–1121

Melia J, Ellman R and Chamberlain J (1994) Investigating changes in awareness about cutaneous malignant melanoma in Britain using the Omnibus Survey. *Clin Exp Dermatol* **19**: 375–379

Melia J, Cooper EJ, Frost T, Graham Brown R, Hunter J, Marsden A, du Vivier A, White J, Whitehead S, Warin AP, et al (1995a) Cancer Research Campaign health education programme to promote the early detection of cutaneous malignant melanoma. I. Work-load and referral patterns. *Br J Dermatol* **132**: 405–413

Melia J, Cooper EJ, Frost T, Graham Brown R, Hunter J, Marsden A, du Vivier A, White J, Whitehead S, Warin AP, Wroughton M, Ellman R and Chamberlain J (1995b) Cancer Research Campaign health education programme to promote the early detection of cutaneous malignant melanoma. II. Characteristics and incidence of melanoma. *Br J Dermatol* **132**: 414–421

Melia J, Harland C, Moss S, Eiser JR and Pendry L (2000) Feasibility of targeted early detection for melanoma: a population based screening study. *Br J Cancer* **82**: 1605–1609

Moss SM, Coleman DA, Chamberlain J, Mapp TJ and for the United Kingdom Trial of Early Detection of Breast Cancer Group (1999) 16-year mortality from breast cancer in the UK Trial of Early Detection of Breast Cancer. *Lancet* **353**: 1909–1914

Office for National Statistics (1998) *Mortality Statistics 1997*. Series DH2 Ed.

Osborne JE, Bourke JF, Holder J, Colloby P and Graham-Brown RA (1998) The effect of the introduction of a pigmented lesion clinic on the interval between referral by family practitioner and attendance at hospital. *Br J Dermatol* **138**: 418–421

Severi G, Giles GG, Robertson C, Boyle P and Autier P (2000) Mortality from cutaneous melanoma: evidence for contrasting trends between populations. *Br J Cancer* **82**: 1887–1891

Sharp S and Sterne J (1997) sbe 16: Meta-analysis. *Stata Technical Bulletin* **38**: 9–14

Sinclair R (1998) Commentary: Start with the KISS principle. *BMJ* **316**: 38–39

Streetly A and Markowe H (1995) Changing trends in the epidemiology of malignant melanoma: gender differences and their implications for public health. *Int J Epidemiol* **24**: 897–907

Tabar L, Fagerberg CJ, Gad A, Baldetorp L, Holmberg LH, Grontoft O, Ljunquist U, Lundstrom B, Manson JC, Eklund G and Day NE (1985) Reduction in mortality from breast cancer after mass screening with mammography. Randomised trial from the Breast Cancer Screening Working Group of the Swedish National Board of Health and Welfare. *Lancet* **i**: 829–832

van der Rhee HJ, van der Spek-Keijser LMT, Van Westering R and Coebergh JWW (1999) Increase in and stabilization of incidence and mortality of primary cutaneous malignant melanoma in western Netherlands, 1980–95. *Br J Dermatol* **140**: 463–467

Whitehead SM, Wroughton MA, Elwood JM, Davison J and Stewart M (1989) Effects of a health education campaign for the earlier diagnosis of melanoma. *Br J Cancer* **60**: 421–425

Williams HC, Smith D and Du Vivier AWP (1990) Evaluation of public education campaigns in cutaneous melanoma: The King's College Hospital experience. *Br J Dermatol* **123**: 85–92

*© 2001 Cancer Research Campaign*

## References

[PDF_00442] Berwick M., Begg C. B., Fine J. A., Roush G. C., Barnhill R. L. (1996). Screening for cutaneous melanoma by skin self-examination.. J Natl Cancer Inst.

[PDF_00444] Burton R. C., Armstrong B. K. (1994). Recent incidence trends imply a nonmetastasizing form of invasive melanoma.. Melanoma Res.

[PDF_00446] Burton R. C., Coates M. S., Hersey P., Roberts G., Chetty M. P., Chen S., Hayes M. H., Howe C. G., Armstrong B. K. (1993). An analysis of a melanoma epidemic.. Int J Cancer.

[PDF_00449] Cohn-Cedermark G., Månsson-Brahme E., Rutqvist L. E., Larsson O., Johansson H., Ringborg U. (2000). Trends in mortality from malignant melanoma in Sweden, 1970-1996.. Cancer.

[PDF_00452] Cook M. G., Clarke T. J., Humphreys S., Fletcher A., McLaren K. M., Smith N. P., Stevens A., Theaker J. M., Melia J. (1996). The evaluation of diagnostic and prognostic criteria and the terminology of thin cutaneous malignant melanoma by the CRC Melanoma Pathology Panel.. Histopathology.

[PDF_00456] Dennis L. K. (1999). Analysis of the melanoma epidemic, both apparent and real: data from the 1973 through 1994 surveillance, epidemiology, and end results program registry.. Arch Dermatol.

[PDF_00459] Doherty V. R., MacKie R. M. (1988). Experience of a public education programme on early detection of cutaneous malignant melanoma.. BMJ.

[PDF_00461] Edman R. L., Klaus S. N. (2000). Is routine screening for melanoma a benign practice?. JAMA.

[PDF_00463] Giles G. G., Armstrong B. K., Burton R. C., Staples M. P., Thursfield V. J. (1996). Has mortality from melanoma stopped rising in Australia? Analysis of trends between 1931 and 1994.. BMJ.

[PDF_00466] Healsmith M. F., Bourke J. F., Osborne J. E., Graham-Brown R. A. (1994). An evaluation of the revised seven-point checklist for the early diagnosis of cutaneous malignant melanoma.. Br J Dermatol.

[PDF_00469] Herd R. M., Cooper E. J., Hunter J. A., McLaren K., Chetty U., Watson A. C., Gollock J. (1995). Cutaneous malignant melanoma. Publicity, screening clinics and survival--the Edinburgh experience 1982-90.. Br J Dermatol.

[PDF_00472] Jackson A., Wilkinson C., Ranger M., Pill R., August P. (1998). Can primary prevention or selective screening for melanoma be more precisely targeted through general practice? A prospective study to validate a self administered risk score.. BMJ.

[PDF_00476] La Vecchia C., Lucchini F., Negri E., Levi F. (1999). Recent declines in worldwide mortality from cutaneous melanoma in youth and middle age.. Int J Cancer.

[PDF_00479] Lipsker D. M., Hedelin G., Heid E., Grosshans E. M., Cribier B. J. (1999). Striking increase of thin melanomas contrasts with stable incidence of thick melanomas.. Arch Dermatol.

[PDF_00482] Little P., Keefe M., White J. (1995). Self screening for risk of melanoma: validity of self mole counting by patients in a single general practice.. BMJ.

[PDF_00485] MacKie R. M., Hole D. (1992). Audit of public education campaign to encourage earlier detection of malignant melanoma.. BMJ.

[PDF_00487] MacKie R. M., Hole D., Hunter J. A., Rankin R., Evans A., McLaren K., Fallowfield M., Hutcheon A., Morris A. (1997). Cutaneous malignant melanoma in Scotland: incidence, survival, and mortality, 1979-94. The Scottish Melanoma Group.. BMJ.

[PDF_00493] Melia J., Cooper E. J., Frost T., Graham-Brown R., Hunter J., Marsden A., Du Vivier A., White J., Whitehead S., Warin A. P. (1995). Cancer Research Campaign health education programme to promote the early detection of cutaneous malignant melanoma. I. Work-load and referral patterns.. Br J Dermatol.

[PDF_00498] Melia J., Cooper E. J., Frost T., Graham-Brown R., Hunter J., Marsden A., Du Vivier A., White J., Whitehead S., Warin A. P. (1995). Cancer Research Campaign health education programme to promote the early detection of cutaneous malignant melanoma. II. Characteristics and incidence of melanoma.. Br J Dermatol.

[PDF_00490] Melia J., Ellman R., Chamberlain J. (1994). Investigating changes in awareness about cutaneous malignant melanoma in Britain using the Omnibus Survey.. Clin Exp Dermatol.

[PDF_00503] Melia J., Harland C., Moss S., Eiser J. R., Pendry L. (2000). Feasibility of targeted early detection for melanoma: a population-based screening study.. Br J Cancer.

[PDF_00511] Osborne J. E., Bourke J. F., Holder J., Colloby P., Graham-Brown R. A. (1998). The effect of the introduction of a pigmented lesion clinic on the interval between referral by family practitioner and attendance at hospital.. Br J Dermatol.

[PDF_00515] Severi G., Giles G. G., Robertson C., Boyle P., Autier P. (2000). Mortality from cutaneous melanoma: evidence for contrasting trends between populations.. Br J Cancer.

[PDF_00520] Streetly A., Markowe H. (1995). Changing trends in the epidemiology of malignant melanoma: gender differences and their implications for public health.. Int J Epidemiol.

[PDF_00523] Tabár L., Fagerberg C. J., Gad A., Baldetorp L., Holmberg L. H., Gröntoft O., Ljungquist U., Lundström B., Månson J. C., Eklund G. (1985). Reduction in mortality from breast cancer after mass screening with mammography. Randomised trial from the Breast Cancer Screening Working Group of the Swedish National Board of Health and Welfare.. Lancet.

[PDF_00532] Whitehead S. M., Wroughton M. A., Elwood J. M., Davison J., Stewart M. (1989). Effects of a health education campaign for the earlier diagnosis of melanoma.. Br J Cancer.

[PDF_00535] Williams H. C., Smith D., du Vivier A. W. (1990). Evaluation of public education campaigns in cutaneous melanoma: the King's College Hospital experience.. Br J Dermatol.

[PDF_00528] van der Rhee H. J., van der Spek-Keijser L. M., van Westering R., Coebergh J. W. (1999). Increase in and stabilization of incidence and mortality of primary cutaneous malignant melanoma in Western Netherlands, 1980-95.. Br J Dermatol.

